# Pilot trials in physical activity journals: a review of reporting and editorial policy

**DOI:** 10.1186/s40814-018-0317-1

**Published:** 2018-07-17

**Authors:** Elsie Horne, Gillian A. Lancaster, Rhys Matson, Ashley Cooper, Andy Ness, Sam Leary

**Affiliations:** 1 National Institute for Health Research Bristol Biomedical Research Centre, University Hospitals Bristol NHS Foundation Trust and University of Bristol, 3rd Floor, Education & Research Centre, Upper Maudlin Street, Bristol, BS2 8AE UK; 2 Research Institute for Primary Care & Health Sciences, Keele University, Staffordshire, ST5 5BG UK; 3 Centre for Exercise, Nutrition and Health Sciences, School for Policy Studies, University of Bristol, 8 Priory Road, Bristol, BS8 1TZ UK

**Keywords:** Literature review, Pilot trial, Feasibility study, Physical activity, Editorial policy

## Abstract

**Background:**

Since the early 2000s, a number of publications in the medical literature have highlighted inadequacies in the design, conduct and reporting of pilot trials. This work led to two notable publications in 2016: a conceptual framework for defining feasibility studies and an extension to the CONSORT 2010 statement to include pilot trials. It was hoped that these publications would educate researchers, leading to better use of pilot trials and thus more rigorously planned and informed randomised controlled trials. The aim of the present work is to evaluate the impact of these publications in the field of physical activity by reviewing the literature pre- and post-2016. This first article presents the pre-2016 review of the reporting and the current editorial policy applied to pilot trials published in physical activity journals.

**Methods:**

Fourteen physical activity journals were screened for pilot and feasibility studies published between 2012 and 2015. The CONSORT 2010 extension to pilot and feasibility studies was used as a framework to assess the reporting quality of the studies. Editors of the eligible physical activity journals were canvassed regarding their editorial policy for pilot and feasibility studies.

**Results:**

Thirty-one articles across five journals met the eligibility criteria. These articles fell into three distinct categories: trials that were carried out in preparation for a future definitive trial (23%), trials that evaluated the feasibility of a novel intervention but did not explicitly address a future definitive trial (23%) and trials that did not have any clear objectives to address feasibility (55%). Editors from all five journals stated that they generally do not accept pilot trials, and none gave reference to the CONSORT 2010 extension as a guideline for submissions.

**Conclusion:**

The result that over half of the studies did not have feasibility objectives is in line with previous research findings, demonstrating that these findings are not being disseminated effectively to researchers in the field of physical activity. The low standard of reporting across most reviewed articles and the neglect of the extended CONSORT 2010 statement by the journal editors highlight the need to actively disseminate these guidelines to ensure their impact.

**Electronic supplementary material:**

The online version of this article (10.1186/s40814-018-0317-1) contains supplementary material, which is available to authorized users.

## Background

Pilot trials play a crucial role in the design of randomised controlled trials (RCT). They provide an opportunity to identify and address feasibility issues prior to the main RCT, thus avoiding the wasted resources and unnecessary participant burden that can incur from poorly designed RCTs. However, there is some confusion in the research community over the definition, purpose, conduct and reporting of pilot studies. A number of publications describe the tendency for small underpowered studies which focus on testing efficacy or effectiveness to be inappropriately described by authors as pilot or feasibility studies [1–4].

In response to these findings, 2016 saw the release of two notable publications that aimed to address the inadequacies and misunderstandings surrounding pilot and feasibility work. The first, published in March 2016, addressed the inconsistencies in the use of the terms pilot and feasibility across medical literature [5]. This publication presented a conceptual framework for defining feasibility and pilot studies in preparation for RCTs. The authors concluded that feasibility is an overarching term that asks whether something will work. A feasibility study asks whether something can be done, should we proceed with it and if so how. A pilot study is a study in which a part or a whole of a future study is conducted on a smaller scale to see whether it will work. Therefore, all pilot studies are feasibility studies, but not all feasibility studies are pilot studies. To clarify, a study in which participants fill in a questionnaire to assess the types of outcomes that they think are important is given by Eldridge et al. as an example of a feasibility study which is not a pilot study [5].

The second paper, published in September 2016, presented a Consolidated Standards of Reporting (CONSORT) 2010 statement extension to include randomised pilot and feasibility trials carried out in advance of a future definitive trial [6]. For brevity, we will refer to this publication as the CONSORT 2010 extension. Eldridge et al. use the term *pilot trial* to refer *to* “any randomised study in which a future definitive RCT, or a part of it, is conducted on a smaller scale” [6]. We will use the term *pilot trial* to refer to any article that fits the inclusion criteria outlined in our methods section. In theory, this should be consistent with the terminology used by Eldridge et al.

While these two publications have the potential to mark a turning point in the conduct, reporting and publication of feasibility work, it is important to evaluate the impact. The challenges and uncertainties faced when carrying out a trial can vary depending on the area of research, making it informative to evaluate the impact of these guidelines in specific fields. Physical activity is a growing field of research due to its associations with some of the most prevalent morbidities in western society, such as type 2 diabetes, cardiovascular disease and certain cancers [7, 8]. Some examples of uncertainties and challenges faced in this field include recruiting hard-to-reach individuals [9], measuring physical activity in a free-living setting (physical activity carried out in a participant’s own environment at their own pace) [10] and initiating and maintaining behaviour change, particularly in older people [11]. It is an essential pre-requisite to the development of effective physical activity interventions that the uncertainties surrounding definitive trials are appropriately addressed in advance by well-conducted pilot trials. Furthermore, pilot trials should be reported in a transparent manner to inform other researchers in the field.

The overall aim of this work is to evaluate the impact of the CONSORT 2010 extension in the field of physical activity. This will be done by reviewing the reporting of pilot trials in physical activity journals before and after the 2016 publication of the CONSORT 2010 extension. This first article presents a review of articles published in 2012–15. Our intention is to carry out a follow-up review of articles published in 2018–21 to evaluate the impact. The objectives of both will be to review the reporting and methodological components of external randomised pilot trials across a selection of physical activity journals and to review the editorial policy regarding the publication of pilot and feasibility trials across these journals.

## Methods

### Identification of articles

Our initial review was carried out across 14 journals (Table 1) concerned with physical activity, exercise and sport. These 14 journals were intentionally generic and not specific to conditions or populations, for example we included the Journal of Physical Activity and Health but excluded the Journal of Physical Activity and Ageing. MEDLINE was searched for articles with either *randomised* or *randomized*, and either *pilot* or feas* in the title or abstract, restricting the search to the years 2012–15 and the 14 generic physical activity journals. Articles were eligible if they fulfilled either of the following two criteria: they identified as either a *pilot* or *feasibility* study in the title OR they explicitly identified as a pilot or feasibility study in the abstract or introduction (e.g. “This pilot/feasibility study…”). Articles were excluded if they were either not randomised or they reported an internal pilot trial. Articles from journals with five or more eligible articles were included in the literature review, and these journals were included in the review of editorial policy.

**Table 1:** Physical activity journals

1	Medicine & Science in Sports & Exercise
2	British Journal of Sports Medicine
3	Sports Medicine
4	American Journal of Sports Medicine
5	International Journal of Behavioral Nutrition and Physical Activity
6	Journal of Science and Medicine in Sport
7	Journal of Physical Activity and Health
8	Scandinavian Journal of Medicine & Science in Sports
9	Journal of Sports Medicine
10	Journal of Sports Medicine and Physical Fitness
11	Journal of Sports Science and Medicine
12	Journal of Sports Sciences
13	Clinical Journal of Sport Medicine
14	European Journal of Sport Science

### Data extraction

A data extraction form was developed using the CONSORT 2010 extension as a guide [6]. The development of the form was an iterative process that involved two reviewers piloting it on three articles and updating the form according to disagreements in responses. Data were extracted from each article by two independent reviewers. We did not extract data corresponding to every item on the CONSORT 2010 extension, but instead focused on the items that address features which have been identified by previous research as the main shortcomings of pilot trials [2, 4, 5]. Briefly, these features are the justification of the pilot trial as an assessment of the feasibility of a future definitive trial and the inappropriate use of hypothesis testing in pilot trials. The included CONSORT 2010 extension items are detailed in Table 2.

**Table 2:** CONSORT 2010 extension [6] items corresponding to extracted data

Item number	Checklist item
Title and abstract	
1a	Identification as a pilot or feasibility randomised trial in the title
Introduction	
2a	Scientific background and explanation of rationale for future definitive trial and reasons for randomised pilot trial
2b	Specific objectives or research questions for pilot trial
Methods	
3a	Description of pilot trial design (such as parallel, factorial) including allocation ratio
5	The interventions for each group with sufficient details to allow replication, including how and when they were actually administered
6a	Completely defined pre-specified assessments or measurements to address each pilot trial objective specified in 2b, including how and when they were assessed
6c	If applicable, pre-specified criteria used to judge whether, or how, to proceed with future definitive trial
7a	Rationale for numbers in trial
12a	Methods used to address each pilot trial objective whether qualitative or quantitative
Discussion	
20	Pilot trial limitations, addressing sources of potential bias and remaining uncertainty about feasibility
21	Generalisability (applicability) of pilot trial methods and findings to future definitive trial and other studies
22a	Implications for progression from pilot trial to future definitive trial, including any proposed amendments

#### Title and abstract

Inconsistencies in the use of the terms *pilot* and *feasibility* have been highlighted by previous publications [2, 12]. This issue was addressed by Eldridge et al. in their development of a conceptual framework for defining feasibility trials in preparation for RCTs, published in 2016 [5]. This motivated the extraction of data related to *item 1a*. Contrasting the use of the terms *pilot* and *feasibility* before and after this publication provides the opportunity to evaluate whether it affected terminology in this field.

#### Introduction

To assess adherence to *item 2a*, we recorded whether the article gave rationale for the future definitive trial and rationale for carrying out a pilot trial. Corresponding to *item 2b*, we recorded whether the article gave clear objectives to assess the feasibility of a future definitive trial.

#### Methods

To investigate the design of the pilot trials in our review, we extracted data corresponding to *items* 3a and *5*. To address *item 3a*, we categorised the articles into the following groups, based on their design: parallel, crossover, cluster, waitlist control and other. The inclusion of a control group is not mandatory in pilot trials; a control group should only be included if it is necessary for addressing uncertainties regarding the future definitive RCT. We recorded whether the trial included a control group, corresponding to *item 5*.

Pilot trial objectives should address the feasibility of a future definitive trial, making *items 6a* and *6c* key to the appropriate reporting of a pilot trial. The outcomes to address these objectives should be completely defined and pre-specified, as per *item 6a*. Each outcome should correspond to a specific aspect of feasibility being addressed by the pilot trial.

Thabane et al. [1] proposed four aspects to broadly classify the different rationale for performing a pilot trial; full details on these aspects can be found in their paper. Briefly, the four aspects are *process* (e.g. recruitment and retention rates), *resources* (e.g. cost, time, equipment), *management* (e.g. data entry and storage) and *scientific* (e.g. dose). We used this classification, including two further categories, to explain the aspects of feasibility addressed by the pilot trials. The two further categories added were *sample size* (pilot trial used to inform the sample size calculation for future definitive trial) and *feedback* (pilot trial used to collect qualitative or quantitative feedback from participants and staff, e.g. to explore the acceptability of the intervention or suggestions for improvements).

For an article to qualify as having addressed any of these aspects of feasibility, the aspect had to be addressed explicitly as an objective in the introduction or as an outcome in the methods section. If applicable, pre-specified criteria should be applied to these outcomes in order to inform the progression to a future definitive trial, corresponding to *item 6c*.

While a formal sample size calculation is not a requirement in pilot trials, the article should give a rationale for the number of participants in the trial, corresponding to *item 7a*. We extracted two pieces of information regarding sample size from each article. The first was whether the study gave rationale for the number of participants in the study, based on the numbers required to assess feasibility of the future definitive trial. The second was whether a sample size calculation had been carried out, based on hypothesis testing of the primary outcome intended for the future definitive trial. The latter refers to the type of sample size calculation that should be carried out in a definitive RCT, whose primary objective is to assess the effectiveness of an intervention.

A previous literature review identified that pilot trials put inappropriate emphasis on hypothesis testing [2]. The CONSORT 2010 extension explains in reference to *item 12a* that “any estimates of effect using participant outcomes as they are likely to be measured in the future definitive RCT would be reported as estimates with 95% confidence intervals without p-values” [6]*.* Corresponding to this item, we recorded whether hypothesis-testing of effectiveness was carried out.

#### Discussion

Discussions of pilot trials have been shown to be particularly poorly reported [4]. Shanyinde et al. highlight that discussions of pilot trials often focus on efficacy, rather than feasibility issues or the planning of future trials [4]. In line with this, it is important to distinguish *items 20* and *21* in the CONSORT 2010 [6] extension to pilot trials to *items 20* and *21* in the CONSORT 2010 statement for definitive RCTs [13].

The “limitations and sources of potential bias” part of *item 20* (CONSORT 2010 extension) should be considered in reference to the progression to a future definitive RCT, and how the design could be altered to overcome them. Similarly, the generalisability referred to in *item 21* (CONSORT 2010 extension) should be considered in the context of generalisability of findings and methods to a future definitive RCT, rather than generalisability of findings to a clinical setting, as is the case when discussing the findings of a definitive RCT. The information extracted corresponding to these items is presented under the following headings: sources of potential bias, remaining uncertainty about feasibility and generalisability to future definitive trial.

As pilot trials should be carried out primarily to assess feasibility of a future definitive RCT, the implications for a future definitive RCT should be made clear in the discussion of the pilot trial, as per *item 22a*. We extracted information regarding the implications for a future definitive RCT, the planned progression to a future definitive RCT and the realised progression to a future definitive RCT. Planned progression was categorised as future definitive RCT planned without any changes, planned with changes from the pilot trial, not planned because of major problems with feasibility or unclear. The information for realised progression to future definitive trial was obtained by an online search as a first step, and where this did not produce results, we contacted the first author of each article by email to request the information. Realised progression was categorised as definitive RCT completed, definitive RCT registered, definitive RCT not registered or no information (if both our online search was unsuccessful and we did not receive a response to the email enquiry).

### Editorial policy

Editors of the physical activity journals with five or more eligible articles received an email enquiry regarding their editorial policy for pilot trials. All editors were sent an initial email and a follow-up email 1 month later if they did not respond. Their responses, along with the information provided on the journal website, are included in this review.

## Results

**Fig. 1:**
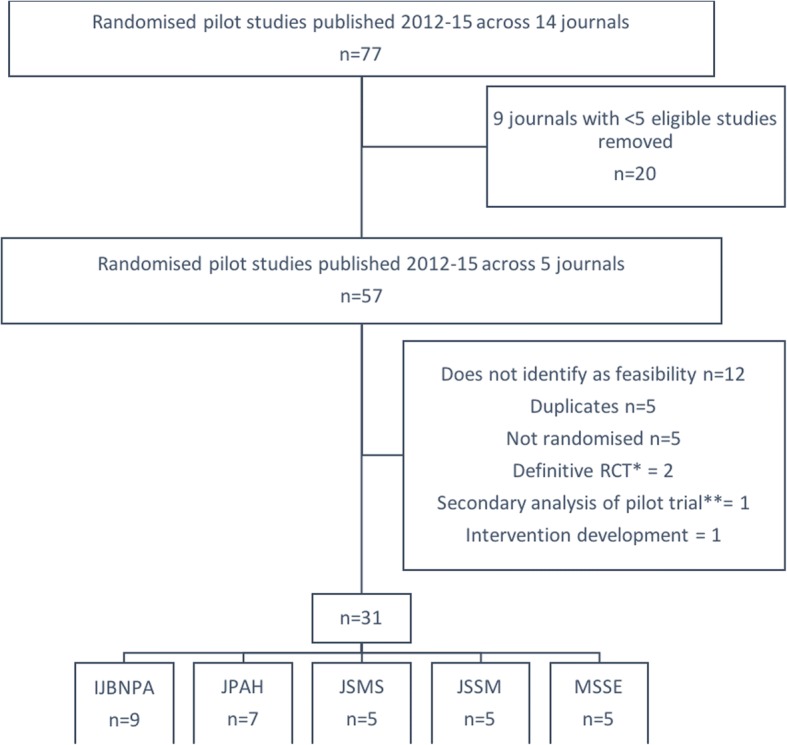
Flow of articles into review. *With outcomes looking at the feasibility of translation to practice. **Exploring mediators of intervention effect. IJBNPA = International Journal of Behavioral Nutrition and Physical Activity; JPAH = Journal of Physical Activity and Health; JSMS = Journal of Science and Medicine in Sport; JSSM = Journal of Sports Science and Medicine; MSSE = Medicine & Science in Sport & Exercise

Figure 1 illustrates the flow of articles into the review and names the included journals. The initial search across 14 journals identified 77 articles. Restricting to journals with five or more relevant articles left 57 articles across five journals. After further exclusions, 31 articles across five journals were included in the review (Table 3).

**Table 3:** Articles included in literature review

Author	Year	Journal	Title	Participants randomised (*n*)	Type
Anderson et al. [14]	2014	IJBNPA	Breast cancer risk reduction—is it feasible to initiate a randomised controlled trial of a lifestyle intervention programme (ActWell) within a national breast screening programme?	80	FDT
Barnes et al. [22]	2015	JPAH	Feasibility and preliminary efficacy of the AMDE4Life program: a pilot randomised controlled trial	48	FNI
Baruth et al. [24]	2015	JPAH	Effects of home-based walking on quality of life and fatigue outcomes in early stage breast cancer survivors: a 12-week pilot study	32	NF
Chau et al. [36]	2014	IJBNPA	The effectiveness of sit-stand workstations for changing office workers’ sitting time: results from the Stand@Work randomised controlled trial pilot	42	NF
Ciolac et al. [18]	2015	MSSE	Prescribing and regulating exercise with RPE after heart transplant: a pilot study	15	NF
Currie et al. [37]	2015	JSMS	Effects of resistance training combined with moderate-intensity endurance or low-volume high-intensity interval exercise on cardiovascular risk factors in patients with coronary artery disease	19	NF
Filingeri et al. [20]	2012	JSSM	The effects of vibration during maximal graded cycling exercise: a pilot study	12	NF
Gatterer et al. [16]	2014	JSSM	Shuttle-run sprint training in hypoxia for youth elite soccer players: a pilot study	16	FNI
Gavin et al. [19]	2014	JSMS	Reproducibility of lactate markers during 4 and 8 min stage incremental running: a pilot study	19	NF
Gennuso et al. [25]	2013	JPAH	Resistance training congruent with minimal guidelines improves function in older adults: a pilot study	25	NF
Gorgey et al. [38]	2012	MSSE	Effects of resistance training on adiposity and metabolism after spinal cord injury	9	NF
Grey et al. [26]	2013	IJBNPA	Weight management for overweight and obese men delivered through professional football clubs: a pilot randomised trial	103	FDT
Greaves et al. [30]	2015	IJBNPA	Waste the waist: a pilot randomised controlled trial of a primary care based intervention to support lifestyle change in people with high cardiovascular risk	108	FDT
Headley et al. [39]	2012	MSSE	Exercise training improves HR responses and VO2peak in predialysis kidney patients	21	NF
Hutchison et al. [40]	2015	JSMS	The effect of footwear and foot orthoses on transverse plane knee motion during running—a pilot study	14	NF
Jago et al. [27]	2013	IJBNPA	Feasibility trial evaluation of a physical activity and screen-viewing course for parents of 6 to 8-year-old children: teamplay	75	FDT
Jago et al. [41]	2014	IJBNPA	Randomised feasibility trial of a teaching assistant led extracurricular physical activity intervention for 9- to 11-year olds: action 3:30	539	FDT
Jago et al. [42]	2012	IJBNPA	Bristol girls dance project feasibility trial: outcome and process evaluation results	210	FDT
Kass et al. [43]	2013	JSSM	A pilot study on the effects of magnesium supplementation with high and low habitual dietary magnesium intake on resting and recovery from aerobic and resistance exercise and systolic blood pressure	16	NF
Kong et al. [17]	2014	MSSE	A pilot walking program promotes moderate-intensity physical activity during pregnancy	37	FNI
Mark et al. [28]	2013	JPAH	Testing the effectiveness of exercise videogame bikes among families in the home-setting: a pilot study	30	NF
Martens et al. [44]	2012	JPAH	The short-term efficacy of a brief motivational intervention designed to increase physical activity among college students	70	NF
Martin et al. [23]	2013	JSMS	Improving muscular endurance with the Mve fitness chair in breast cancer survivors: a feasibility and efficacy study	26	FNI
Riley et al. [29]	2015	JSMS	Outcomes and process evaluation of a programming integrating physical activity into the primary school mathematics curriculum: the EASY Minds pilot randomised controlled trial	54	FNI
Rogers et al. [45]	2014	MSSE	Biobehavioural factors mediate exercise effects on fatigue in breast cancer survivors	46	NF
Steeves et al. [46]	2012	IJBNPA	Can sedentary behaviour be made more active? A randomised pilot study of TV commercial stepping versus walking	58	FNI
Suh et al. [21]	2015	JPAH	Pilot trial of a social cognitive theory-based physical activity intervention delivered by non-supervised technology in persons with multiple sclerosis	68	NF
Thogersen-Ntoumani et al. [15]	2014	JPAH	Step by step: the feasibility of a 16-week workplace lunchtime walking intervention for physically inactive employees	75	FNI
Voglar et al. [47]	2014	JSSM	Kinesio taping in young health subjects does not affect postural reflex reactions and anticipatory postural adjustments of the trunk: a pilot study	12	NF
Volaklis et al. [48]	2015	JSSM	Acute pro- and anti-inflammatory responses to resistance exercise in patients with coronary artery disease: a pilot study	8	NF
Yardley et al. [49]	2014	IJBNPA	Randomised controlled feasibility trial of a web-based weight management intervention with nurse support for obese patients in primary care	179	FDT

*IJBNPA* International Journal of Behavioural Nutrition and Physical Activity; *JPAH* Journal of Physical Activity and Health; *MSSE* Medicine & Science in Sport & Exercise; *JSMS* Journal of Science and Medicine in Sport; *JSSM* Journal of Sports Science and Medicine; *FDT* future definitive trial; *FNI* feasibility of novel intervention; *NF* non-feasibility

### Pilot trial categories

After data extraction, it was apparent that the pilot trials in the review could be classified into three categories: trials that were carried out in preparation for a future definitive trial (FDT); trials that evaluated the feasibility of a novel intervention but did not explicitly address a future definitive trial in their objectives (FNI); and trials that had no objectives, pre-defined assessments or measurements to assess feasibility, referred to as non-feasibility (NF). Articles reporting FDT and FNI trials were assessed according to their adherence to all items listed in Table 2. As the NF trials did not address objectives relating to feasibility, these articles were excluded from the sections of the review that focus on the aspects of feasibility addressed and the discussion points that focus on the feasibility of a future definitive trial. The category assigned to each article is given in Table 3. All results are stratified by these three categories (FDT, FNI or NF).

Of the 31 trials included in the review, seven (23%) were FDT, seven (23%) were FNI and 17 (55%) were NF. An interesting observation taken from Table 3 was that all seven of the FDT articles were published in IJBNPA. The FNI and NF articles both had a fairly even distribution across the five journals. In terms of the numbers of participants randomised in each trial, the FDT trials were substantially larger than the FNI and FN trials, with a median of 108 (IQR 130) participants. The FNI trials had a median of 48 (IQR 32) participants, and NF trials were the smallest, with a median of 19 (IQR 24).

### Literature review

The full results for the literature review are detailed in Table 4. All data extracted in this review can be found in Additional file 1.

**Table 4:** Results from data extraction stratified by category of pilot trial (results given as number (%) unless otherwise specified)

Item	Description	Feasibility of future definitive trial (*n* = 7)	Feasibility of novel intervention (*n* = 7)	Non-feasibility (*n* = 17)	Total (*n* = 31)
1a	Words in title				
	Pilot	2 (28)	5 (71)	12 (71)	19 (61)
	Feasibility	5 (71)	3 (43)	0 (0)	8 (26)
	Randomised	5 (71)	3 (43)	1 (6)	9 (29)
2a	Introduction				
	Rationale for future definitive trial	7 (100)	7 (100)	17 (100)	31 (100)
	Rationale for pilot trial	0 (0)	0 (0)	0 (0)	0 (0)
2b	Objectives				
	Objectives to assess the feasibility of a future definitive trial	7 (100)	0 (0)	0 (0)	7 (23)
3a	Design				
	Parallel	4 (57)	5 (71)	8 (47)	17 (55)
	Crossover	0 (0)	0 (0)	6 (35)	6 (19)
	Cluster	2 (29)	0 (0)	0 (0)	2 (6)
	Waitlist control	1 (14)	1 (14)	1 (6)	3 (10)
	Other	0 (0)	1 (14)	2 (12)	3 (10)
5	Treatment groups				
	Median number of treatment groups (IQR)	2 (0)	2 (0)	2 (0)	2 (0)
	Control group	7 (100)	5 (71)	11 (65)	23 (74)
6a	Aspects of feasibility addressed				
	Process (e.g. recruitment and retention rates)	7 (100)	4 (57)	–	11 (35)*
	Resources (e.g. cost, time, equipment)	4 (57)	0 (0)	–	4 (13)*
	Management (e.g. data entry and storage)	0 (0)	0 (0)	–	0 (0)*
	Scientific (e.g. dose)	7 (100)	6 (86)	–	13 (42)*
	Sample size (inform sample size in future definitive trial)	4 (57)	0 (0)	–	4 (13)*
	Feedback (qualitative or quantitative feedback from participants and staff)	7 (100)	5 (71)	–	12 (39)*
	Median number of aspects addressed by each trial (IQR)	4 (1)	2 (2)	–	3 (2)*
6c	Criteria for progression to future definitive trial	0 (0)	0 (0)	–	0 (0)*
7a	Participant numbers				
	Sample size calculation	1 (14)	0 (0)	3 (18)	4 (13)
	Rationale for sample size	4 (57)	1 (14)	–	5 (36)*
12a	Analysis				
	Hypothesis testing	3 (43)	7 (100)	16 (94)	26 (84)
20 and 21	Discussion				
	Sources of potential bias	2 (29)	2 (29)	4 (24)	8 (26)
	Remaining uncertainty about feasibility	5 (71)	1 (14)	–	6 (43)*
	Generalisability to future definitive trial	3 (43)	0 (0)	–	3 (21)*
22a	Implications for future definitive trial	7 (100)	3 (43)	–	10 (71)*
	Progression planned				
	Definitive RCT planned without any changes	0 (0)	0 (0)	–	0 (0)*
	Definitive RCT planned with changes from the pilot trial	6 (86)	1 (14)	–	7 (50)*
	Definitive RCT not planned because of major problems with feasibility	1 (14)	0 (0)	–	1 (7)*
	Unclear	0 (0)	6 (86)	–	6 (43)*
	Progression realised				
	Definitive trial completed	3 (43)	0 (0)	–	3 (21)*
	Definitive trial registered	1 (14)	1 (14)	–	1 (7)*
	Definitive trial not registered	0 (0)	2 (29)	–	2 (14)*
	No information	3 (43)	4 (57)	–	8 (57)*

*Percentage of the 14 FDT and FNI trials

#### Words in title

Notable differences were found across the three categories of trials in terms of how they identified in the title of the article. FDT and FNI trials varied as to whether they identified as *pilot* or *feasibility*, all of them identifying as either or both. In contrast, 12 (71%) of the NF trials identified as *pilot* in the title, and none as *feasibility.* In addition, only one (6%) of the NF trials identified as randomised, compared with five (71%) and three (43%) of the FDT and FNI studies respectively. The original CONSORT 2010 statement advises that RCTs should identify as randomised in the title [6], thus highlighting the poor reporting in these trials in general as these were the guidelines they should have been following at the time of publication.

#### Introduction

Across all articles, the introduction focused on the scientific background and rationale for carrying out a definitive trial. However, none of the articles reported uncertainties in the context of relevant evidence in their introduction, and none gave a clear rationale for the need to carry out a pilot trial as opposed to a definitive trial. Even in the trials with feasibility objectives (FDT and FNI), the rationale for exploring feasibility was not supported with relevant evidence.

Specific aspects of feasibility to be addressed were outlined in the introduction in all but one of the FDT articles. This article [14] simply stated “This study aims to assess the feasibility…” with no detail on the specific aspects of feasibility to be addressed. However, the specific aspects of feasibility to be addressed were detailed by Anderson et al., in the methods section of the article [14].

In contrast, only one (14%) FNI article detailed specific aspects of feasibility to be addressed in their introduction [15]. However, five (71%) of the FNI articles did detail aspects of feasibility to be addressed in the methods section. Of the remaining two, one revealed the specific aspect of feasibility that they were addressing in the discussion [16], while one did not address any specific aspects of feasibility, listing only outcomes to address the effectiveness of the intervention [17].

Inherent to the labelling of the NF trials, none of the NF articles detailed feasibility objectives in the introduction or in the methods section.

#### Objectives

None of the NF articles made reference to feasibility when stating their objectives. Thirteen (76%) listed effectiveness of an intervention as their primary or sole objective, and the remaining four listed objectives relating to usefulness of a scale [18], reproducibility of a test [19], monitoring physiological mechanisms [20] and efficacy [21]. None of these articles outlined rationale for the need to carry out a pilot trial as opposed to a definitive trial.

#### Design

Of all 31 studies, a parallel design was used in 55%, crossover in 19%, cluster in 6%, waitlist control in 10% and the remaining 10% had other designs. All six crossover trials were in the NF category, accounting for 35% of the total NF trials.

#### Treatment groups

All but three of the trials were two-arm; of the three that were not, one was three- and two were four-arm. All of the FDT trials had a control arm, while a control arm was included in five (71%) and 11 (65%) of the FNI and NF studies respectively.

#### Aspects of feasibility addressed

The NF articles are omitted as they did not address feasibility. Process, scientific and feedback aspects were addressed by all seven of the FDT trials, and by four (57%), six (86%) and five (71%) of the FNI trials respectively. Resources and sample size were both addressed by four (57%) of the FDT, but by none of the FNI trials. Neither the FDT nor FNI trials addressed management as an aspect of feasibility. The median number of aspects of feasibility addressed was four (IQR one) in the FDT trials, compared with two (IQR one) in the FNI trials.

None of the trials detailed pre-specified criteria used to judge whether, or how, to proceed with a future definitive trial. However, two of the FNI trials specified a minimum attendance rate for the intervention to be deemed feasible [22, 23], but direct implications for a future randomised trial were not detailed.

#### Participant numbers

Rationale for the number of participants in the study was given in four (57%) of the FDT articles and one (14%) of the FNI articles. Of all 31 trials, four (13%) carried out a sample size calculation using the primary outcome intended to test the effectiveness/efficacy of the intervention.

#### Analysis

Hypothesis testing was used in 26 (84%) of the trials in total, despite only four (13%) carrying out sample size calculations to ensure they were powered to do so. The practice of incorporating hypothesis testing into analysis was least prevalent in the FDT trials, but still almost half of these trials did so.

#### Discussion

Only eight (26%) of the total articles addressed sources of potential bias in their discussion [17, 19, 24–29]. Bias should always be addressed when discussing the findings of a trial, regardless of the design, thus highlighting poor reporting of the discussion in general across the articles included in this review. As the remaining three discussion points (corresponding to *items 20*, *21* and *22a* listed in Table 2) refer to the feasibility of a future definitive trial, results are not reported for the NF studies. There was a clear discrepancy between the FDT and FNI articles in the reporting of these discussion points. Remaining uncertainty regarding feasibility and the implications of the pilot trial findings for a future definitive trial were well reported by most FDT articles. However, only three (43%) of the FDT articles reported whether their methods and findings were generalisable to a future definitive trial.

As stated at the beginning of this section, the FNI articles did not explicitly address the feasibility of a future definitive trial in their objectives, instead considering the feasibility of a novel intervention. Only one of the FNI articles addressed the remaining uncertainty about feasibility [15], none considered the generalisability of their methods and findings to a future definitive trial and three (43%) considered the implications of their findings to a future definitive trial. While this highlighted poor reporting of the discussion, it does demonstrate that some of the articles which did not explicitly consider the feasibility of a future definitive trial in their objectives then went on to address it in their discussion.

#### Progression planned/realised

In terms of progression to a future definitive RCT, there was a clear distinction in the quality of reporting between the FDT and FNI trials. All but one of the FDT trials planned to carry out a definitive RCT with changes based on the findings from the pilot trial, while plans for progression were unclear in the remaining one FDT trial. Conversely, plans for progression were unclear in all but one of the FNI trials, with the remaining one stating that a future definitive RCT was planned with changes based on pilot trial findings. None of the studies planned to progress to a future definitive trial without changes. Of the six studies in which the plans were unclear, the lack of clarity generally related to whether the suggested changes were due to be implemented in a future definitive RCT, or whether they should be tested in further feasibility work, prior to carrying out a definitive RCT.

Three (43%) of the FDT trials progressed to definitive trials which have since been completed. One trial was registered [30]. However, following contact with Greaves (the lead author), we understand that the definitive trial does not directly correspond to the pilot trial, as it was carried out in a different region, under a different institution and with additional components, but uses the intervention piloted by Greaves et al. The protocol for the definitive trial has been published [31]. We did not obtain information from the remaining three FDT trials. Of the FNI trials, one had been registered as a definitive trial, two were not registered (although the authors stated intentions to do so when contacted) and we did not obtain any information on the remaining four FNI trials.

### Review of editorial policy

Of the five journals, only the International Journal of Behavioral Nutrition and Physical Activity (IJBNPA) and Journal of Physical Activity and Health (JPAH) detailed their editorial policy for pilot trials on the journal website or in the author guidelines, both stating that they rarely accept pilot trials. Editors from all five journals responded to our enquiry regarding their editorial policy for pilot trials. Across all five journals, the editors stated that they generally do not accept pilot trials, although none stated that they would be automatically rejected without review, thus giving themselves some flexibility. Editors from IJBNPA and JPAH stated that they would only consider pilot studies that were novel and well-reported, while editors from the Journal of Science and Medicine in Sport (JSMS) and the Journal of Sports Science and Medicine (JSSM) did not state any criteria specific to pilot trials and requested only consistency with their author guidelines for research articles.

## Discussion

In agreement with the findings of Shanyinde et al. [4], yet in contrast to those of Arain et al. [2], we found more articles identified as *pilot* than *feasibility* in this subject area. As the term *feasibility* was used only in articles with appropriate feasibility objectives (labelled FDT or FNI in this review), we did not observe the misuse of this term in our review. Conversely, the term *pilot* was used across articles that did not have feasibility objectives but instead tested an intervention’s effectiveness on a small sample and at a single site (labelled NF in this review).

Our review found that, beyond the lack of clear feasibility objectives, the defining characteristics of the trials inappropriately labelled as *pilot* (NF trials) were that they had small sample sizes unsupported by sample size calculation and that they used hypothesis tests despite most being underpowered to do so. Not only is the inappropriate use of the term *pilot* misleading in this context, the conduct of such trials is in most cases unethical, as they put participants at risk for limited benefit [32]. These findings reinforce the need to disseminate the *Conceptual Framework to Define Feasibility and Pilot Studies* [5] to discourage inappropriate use of the term *pilot* and dissuade the practice of conducting a main trial in miniature to test effectiveness*.* Conditional on the dissemination of the *Conceptual Framework to Define Feasibility and Pilot Studies* [5], we anticipate very few, if any, such pilot trials will be identified in the follow-up review of articles published in 2018–21.

It is also of note that 35% of the inappropriately labelled pilot trials (NF trials) were cross-over in design. The benefit of this design is that, by making comparisons within rather than between participants, fewer participants are required to detect a change in the primary outcome compared with the number needed in an equivalent parallel trial [33]. However, this design has a history of inappropriate use, for example, in the field of fertility medicine [34].The motivation for choosing this design should be driven by context, not by low participant numbers. Only one of the six cross-over pilot trials in this review reported a sample size calculation, suggesting that the design could have been motivated by small sample size in the other five cases. The reason for conducting a pilot trial should be to inform a future definitive RCT. Therefore, the use of the cross-over design in pilot trials should be discouraged unless this is the intended design for the future RCT. To elaborate, feasibility issues in the pilot trial may be associated with the cross-over design and thus not applicable to the definitive trial of a different design. To our knowledge, the inappropriate use of the cross-over design in pilot trials has not been highlighted by previous reviews. However, it is likely to be of relevance across other areas of medical research and not only in physical activity.

Amongst the articles with feasibility objectives (FDT and FNI trials), many did not give appropriate reference to the future definitive trial in their introductions and discussions. However, we are hopeful that the publication of the CONSORT 2010 extension should ameliorate this issue, as the guidelines give explicit recommendations to both justify the need for a pilot in advance of a future definitive trial and to discuss the findings in relation to a future definitive trial. At the time of these articles’ publication, no such guidelines existed.

A more concerning practice amongst the articles with feasibility objectives (FDT and FNI trials) was that many gave inappropriate emphasis to hypothesis tests of the primary outcome intended for the definitive trial. In a review published in 2004, Lancaster et al. recommend that the analysis of pilot studies “should be mainly descriptive” and that “results from hypothesis testing should be treated with caution, as no formal power calculations have been carried out” [3]*.* In the follow-up to this review, Arain et al. conclude that pilot studies still put “inappropriate emphasis on hypothesis testing” [2]. This raises the concern that these recommendations are either not reaching the relevant researchers or that they are being ignored. This calls for the need for both better scientific training and better dissemination of research methodology in the field of pilot and feasibility work.

The launch of the *Pilot and Feasibility Studies* journal in 2015 was a major step to address these issues and was described by Lancaster as providing “a forum for discussion of methodological issues that will lead to increased scientific rigour in this area” [12]. The journal also provides a platform for the publication of pilot and feasibility work. Our review of editorial policy, which identified an increasing reluctance to publish pilot work across the five reviewed physical activity journals, emphasises the need for a journal dedicated to the publication of pilot work.

While the multi-disciplinary nature of *Pilot and Feasibility Studies* has the benefit of sharing ideas across different subject areas, it is also crucial that subject-specific journals acknowledge the importance of pilot and feasibility work. This means considering prospective pilot trial submissions on the merit of their potential to inform future research, rather than the significance of an effect size. A key step to implementing these changes in editorial policy is the adoption of the CONSORT 2010 extension as a guideline for submissions identified as *pilot* or *feasibility* work.

To our knowledge, this is the first review to document the reporting and editorial policy of pilot trials in the field of physical activity. A strength of this work was the extensive use of the CONSORT 2010 statement as a framework for the data extraction form. The CONSORT 2010 statement was extended to pilot trials by a research team with expert input from the research community at multiple stages throughout the process. This is described in detail elsewhere [35]. A further strength of this work was the use of two reviewers for data extraction, which enhanced the accuracy and rigour of the review.

A weakness of this review was the small number of studies included with feasibility objectives. While this reflects the necessity of further work to encourage appropriate use of the term *pilot*, limited conclusions can be drawn from the trends observed within the 14 studies with feasibility objectives. We also anticipated that a greater number of physical activity journals would have published at least five pilot trials in 2012–15. This result either reflects the low number of pilot trials being published in physical activity journals generally or suggests that pilot trials of physical activity interventions are being published elsewhere. The 14 journals included in our search cover some of the highest impact physical activity journals, but the review of editorial policy (limited to the five journals included in the review) identified a reluctance to publish pilot trials in these journals. This could be indicative that physical activity researchers are publishing pilot trials in lower impact journals (not included in our review). An alternative explanation is that condition-specific journals are more open to publishing feasibility work (examples of condition-specific journals that relate to physical activity are *Diabetes Care* and the *European Heart Journal*). The second avenue of further work, outlined in the following paragraph, suggests an alternative approach to reviewing the literature which may provide clarity on this issue.

We suggest two avenues for further work. The first avenue is our intention to carry out a follow-up review using articles published in 2018–21. This follow-up review will use the same methods as the current review and will be used to evaluate the impact of the CONSORT 2010 extension in the field of physical activity. A second avenue for further work would be to identify a collection of articles reporting definitive trials in the field of physical activity and to look backwards to find whether appropriate feasibility work was carried out prior to the definitive trial, and if so, where the feasibility work was published, and how it influenced the design of the definitive trial. This approach would focus on the reporting and conduct of trials with feasibility objectives, thus addressing the weakness mentioned in the previous paragraph. Taken together, these two styles of review would give a more complete picture of the use of feasibility work undertaken for physical activity trials. We recommend that researchers in other fields carry out both styles of review in order to gain a thorough understanding of the feasibility work in their field.

## Conclusions

To summarise, the aim of this study was to review the reporting and methodological components of pilot trials published across a selection of physical activity journals, using the CONSORT 2010 extension as a guide. We designed the search criteria to identify external randomised pilot trials, as these are the trials specifically targeted by the CONSORT 2010 extension. We found that despite identifying as randomised pilot trials, over half (55%) of the articles identified by our search criteria did not list objectives relating to the feasibility of conducting a future definitive trial. These findings are not unique to the field of physical activity and agree with the findings of three previous literature reviews, all reporting the frequent use of the terms *pilot* or *feasibility* to inappropriately describe trials with efficacy or effectiveness as their primary aim. In many cases, these trials had no objectives relating to feasibility [2–4].

## Additional file

Additional file 1: Data extracted in literature review. (CSV 8 kb)
